# Therapeutic and antioxidant potential of bionanofactory *Ochrobactrum sp*.-mediated magnetite and zerovalent iron nanoparticles against acute experimental toxoplasmosis

**DOI:** 10.1371/journal.pntd.0011655

**Published:** 2023-10-06

**Authors:** Nehal Nassef Hezema, Marwa Moustafa Eltarahony, Sara Ahmed Abdel Salam

**Affiliations:** 1 Department of Medical Parasitology, Faculty of Medicine, Alexandria University, Alexandria, Egypt; 2 Department of Environmental Biotechnology, Genetic Engineering and Biotechnology Research Institute, City of Scientific Research and Technological Applications, New Borg El-Arab City, Alexandria, Egypt; Creighton University, UNITED STATES

## Abstract

The control of toxoplasmosis, a rampant one health disease, has been focussed on conventional antitoxoplasmic agents with their adverse outcomes, including serious side effects, treatment failure and emergence of drug resistant strains. Nanobiotechnology may provide a strong impetus for versatile alternative therapies against toxoplasmosis. Bionanofactory *Ochrobactrum sp*. strain CNE2 was recruited for the biosynthesis of functionalized magnetite iron nanoparticles (MNPs) and nanozerovalent iron (nZVI) under aerobic and anaerobic conditions and their therapeutic efficacy was evaluated against acute toxoplasmosis in murine model. The formation of self-functionalized spherical nanoparticles varied in size, identity and surface properties were substantiated. Mice were orally administered 20 mg/kg of each formulation on the initial day of infection and continued for seven consecutive days post infection (PI). Parasitological, ultrastructural, immunological, and biochemical studies were performed for assessment of therapeutic activity of biogenic iron nanoparticles (INPs). Parasitologically, MNPs showed the highest antitoxoplasmic efficacy in terms of 96.82% and 91.87% reduction in mean tachyzoite count in peritoneal fluid and liver impression smears, respectively. Lesser percentage reductions were recorded in nZVI-treated infected subgroup (75.44% and 69.04%). In addition, scanning electron microscopy (SEM) examination revealed remarkable reduction in size and extensive damage to the surface of MNPs-treated tachyzoites. MNPs-treated infected mice revealed a statistically significant increase in the serum levels of both interferon gamma (IFN-γ) to 346.2 ± 4.6 pg/ml and reduced glutathione (GSH) to 8.83 ± 0.30 mg/dl that subsequently exerted malondialdehyde (MDA) quenching action. MNPs showed a superior promising antitoxoplasmic activity with respect to both spiramycin (SPI) and nZVI. To best of our knowledge, this is the first study of a bio-safe oral iron nanotherapeutic agent fabricated via an eco-friendly approach that offers promising potential against acute experimental toxoplasmosis.

## Author summary

Toxoplasmosis is transmitted via food, water, and blood-borne zoonosis caused by an obligate intracellular parasite, *Toxoplasma gondii* (*T*. *gondii*). The opportunistic nature and distinct ability of *T*. *gondii* to inhabit almost all nucleated cells of any warm-blooded host makes it an important global public and animal health concern. Toxoplasmosis, a major neglected tropical disease, may lead to a devastating disease especially among immunocompromised patients and infants born to infected mothers. The emergence of resistant strains and development of serious side effects of the gold standard treatment, pyrimethamine and sulfadiazine, deemed as major causes of treatment failure. Besides, these drugs cannot be used during pregnancy because of the deleterious effects on the fetal development. Consequently, the present work focused on utilization of biologically-synthesized iron nanoparticles (INPs) that can cross cell membranes and reach specific targets more easily than multiple drugs. INPs were biosynthesized by an eco-friendly approach under aerobic and anaerobic conditions. Orally administered INPs displayed significant antioxidant and antitoxoplasmic activity and afforded high levels of safety in mice. Collectively, the marked therapeutic activity and the safe nature of biosynthesized INPs enable them to serve as a promising nanotherapeutic antitoxoplasmic agents.

## Introduction

Toxoplasmosis is a ubiquitous one health disease that affects not only the health of human, domestic and wildlife animals but also the ecosystems [1]. It is caused by the opportunistic parasite, *Toxoplasma gondii* (*T*. *gondii*), that exhibits heteroxenous life cycle comprising felines as definitive hosts and homeotherms (rodents and humans) as intermediate hosts [2]. Man contacts the infection by accidental ingestion of oocysts in contaminated food and water, tissue cysts in improperly cooked meat or tachyzoites in unpasteurized milk [3]. Moreover, toxoplasmosis can be transmitted via blood transfusion, organ transplants or vertical transmission [4]. Although, 90% of infected individuals are asymptomatic, newborns and immunocompromised patients may present with serious health problems [4].

To date, the combination of pyrimethamine, sulphonamides and folinic acid is the gold standard treatment for toxoplasmosis [5]. However, pyrimethamine-related myelosuppression and hypersensitivity reactions to sulphonamides were reported [6,7]. Spiramycin (SPI), azithromycin, clarithromycin, atovaquone, and cotrimoxazole, have been found to be effective in treatment of toxoplasmosis [4]. The reported treatment failures of the current licensed drugs may be related to poor compliance (long-term treatment and drug intolerance) or emergence of drug-resistant strains [8]. To overcome this forthcoming alarming situation, the need for novel, facile yet selective therapeutics against toxoplasmosis is an eternal challenge.

Metal-based nanoparticles (NPs) have unprecedentedly revolutionized research in parasitology, owing to their unique physico-chemical properties [9]. Besides antitoxoplasma potential of chemically synthesized metal NPs [10,11], biogenic metal NPs as silver [12], selenium [13], zinc [14] and copper [15] had shown a considerable antitoxoplasmic activity. Iron is an essential micronutrient that plays a vital role in cellular functions of versatile prokaryotic and eukaryotic cells [16,17]. A large body of evidence supports the high reactivity and biocompatibility of iron-based nanoparticles (INPs) as compared to other noble metals such as gold and silver [18]. Microbial synthesis of INPs, is an eco-friendly approach that converts iron (Fe) ions into nanoscale iron particles using enzymes and versatile secondary metabolites [19]. Among INPs, magnetite iron nanoparticles (MNPs) and nanozerovalent iron (nZVI), have been extensively investigated in treatment of infectious diseases.

Magnetite-based NPs, iron oxide NPs, are preferred in biomedicine due to their biocompatibility, superparamagnetic and biodegradable properties [20]. Some MNPs formulations are approved by Food and Drug Administration for their use in treatment of iron deficiency anemia and as magnetic resonance imaging contrast agents [21]. Apart from the advances witnessed in cancer therapeutics, green-synthesized MNPs have been exploited against various pathogenic strains of bacteria and fungi [22,23,24]. Concerning parasites, a recent *in vivo* study had reported an enhanced activity of biogenic MNPs against *Schistosoma mansoni* (*S*. *mansoni*) [25].

nZVI, a composite consisting of Fe (0) core and ferric oxide coating, has the potential to interact with pollutants in the environment and influence the living organisms [26,27]. Besides remediation of environment, green-synthesized nZVI possesses anti-cancer [28], antibacterial [29,30], antifungal [30], and antischistosomal activities [25].

With such a multitude of applications of green-synthesized MNPs and nZVI, they proved their potential as effective anti-schistosomal agents [25]. Leaping the hurdles in developing treatments for toxoplasmosis, the potential antiparasitic activity of these biogenic INPs is worth to be studied. Accordingly, the objective of the current study was to evaluate the therapeutic and antioxidant potential of biogenic MNPs and nZVI against acute experimental toxoplasmosis. The therapeutic activity of biosynthesized NPS was assessed using parasitological, ultrastructural, immunological and biochemical studies against acute toxoplasmosis in a murine model.

## Materials and methods

### I. Ethics statement

Following the Egyptian national regulations for laboratory animal experimentation, the experimental protocol was approved by the Ethics Committee of the Faculty of Medicine, Alexandria University, Egypt (Protocol approval number: 0305745).

### II. Animals

Four to six-week-old male Swiss strain albino mice, weighing 20–25 grams, were obtained from the animal house of the Medical Parasitology Department, Faculty of Medicine, Alexandria University, Egypt. Mice were kept in a suitable healthy rearing environment with standard conditions of light and temperature as well as free access to food and water.

### III. *T*. *gondii* strain and infection

The Virulent RH HXGPRT (-) strain of *T*. *gondii* was maintained in the laboratory of the Medical Parasitology Department, Faculty of Medicine, Alexandria University, Egypt, by serial intraperitoneal (IP) passage of tachyzoites in Swiss strain albino mice every five days [31]. For animal infection, tachyzoites were harvested from peritoneal exudates of infected mice. Then, the tachyzoites were counted by the haemocytometer and injected intraperitoneally at a dose of 2500 tachyzoites/100 μl saline per mouse for induction of acute infection model [32].

### IV. Drugs

Spiramycin (3 M.I.U.) was purchased from local pharmacy. Each tablet was weighed, crushed and dissolved in phosphate buffer saline (PBS). It was given to mice orally at a dose of 400 mg/kg once daily [33].

MNPs and nZVI were biosynthesized in the laboratory of City of Scientific Research and Technological Applications, Alexandria, Egypt. A pilot study was conducted to determine the lowest effective oral dose of MNPs and nZVI capable of decreasing the total tachyzoites burden, and 20 mg/kg/day was selected.

### V. Biosynthesis and characterization of MNPs and nZVI

The bacterial strain, *Ochrobactrum sp*. *CNE2*, was isolated from a freshwater sample collected from Mahmoudia canal, Alexandria, Egypt. The strain *CNE2* was identified by 16S rDNA sequencing as *Ochrobactrum sp*. *CNE2* strain and its gene sequence was deposited in the GenBank under the accession number of MN631047. On optimized production medium, the bacterial inoculum of 0.5 McFarland (approximately 10^8^ CFU/mL) was cultivated and incubated aerobically and anaerobically according to detailed protocol described by Zaki *et al*. [34]. After complete incubation, the bacterial biomass containing INPs was harvested, washed and INPs were extracted, washed, dried and subjected for further characterization.

The optical features with related surface plasmon resonance (SPR), crystalline phase, particle’s identity, purity, and morphological properties of both biosynthesized INPs were determined through a Labomed model UV-Vis double-beam Spectrophotometer, X-ray diffractometer (XRD) (Shimadzu 7000, USA), and transmission electron microscopy (TEM) (JEOL JEM-1230), respectively. Whereas, the analysis of elemental constituents and functional groups was performed by energy dispersive X-ray spectroscopy (EDX) (JEOL JSM-6360LA) and Fourier-transform infrared spectroscopy (FTIR) (Shimadzu FT-IR-8400 S, Japan), respectively. The surface charge, overall INPs size distribution profile in aqueous solution, poly dispersity index (PDI) and their zeta potential (ZP) were assessed using a zeta sizer (Malvern Instrument ZS-Nano, UK) [35].

### VI. Animal grouping and experimental design

Forty-eight mice were divided into 24 serving as non infected group (I) and 24 as infected group (II). Group I was further subdivided into 4 equal subgroups as follows: Subgroup Ia, Non infected non treated; Subgroup Ib, Non infected SPI-treated; Subgroup Ic, Non infected MNPs-treated; and Subgroup Id, Non infected nZVI-treated. Group II was subdivided into 4 equal subgroups as follows; Subgroup IIa, Infected non treated; Subgroup IIb, Infected SPI-treated; Subgroup IIc, Infected MNPs-treated; and Subgroup IId, Infected nZVI- treated.

The dose of the treating drugs was calculated and suspended in 100 μl of PBS per mouse. 100 μl of PBS were orally administrated to mice of the non treated subgroups (Ia and IIa) in the same schedule as treated subgroups. Treatment was initiated on the same day of infection and continued for seven consecutive days. Mice of Group II were infected intraperitoneally with 2500 tachyzoites/100 μl saline per mouse. Animals of all subgroups were sacrificed on the 8^th^ day post infection (PI) for assessment of efficacy of therapy.

### VII. Parasitological study

#### VII.1. Mortality rate

The mortality rate (MR) was estimated at the time of sacrifice on 8^th^ day PI in treated subgroups (Ib, Ic, Id and IIb, IIc, IId) and compared with their corresponding non treated subgroups (Ia and IIa), respectively [36]. The MR% for each subgroup was assessed by the following equation:

$$MR\;(\%)=\frac{Number\;of\;dead\;mice\;on\;the\;day\;of\;sacrifice}{Number\;of\;mice\;at\;the\;beginning\;of\;the\;experiment}\times 100$$

#### VII.2. Parasite count

Parasite burden was estimated on the day of sacrifice by counting the extracellular tachyzoites in the peritoneal fluid (per ml) using a hemocytometer as well as in ten high power fields of Giemsa-stained impression smears of liver of each infected mouse [33,36]. Then, the mean number of tachyzoites in each infected subgroup was calculated. The percentage reduction (%R) in the parasite burden, whether in peritoneal fluid or liver impression smears, was calculated according to the following equation: [37]

$$Percentage\;reduction\;(\%R)=\frac{N-n}{N}\times 100$$

N: Mean tachyzoite count recovered from the infected non treated control subgroup

n: Mean tachyzoite count recovered from each infected treated subgroup

### VIII. Ultrastructural study

Tachyzoites harvested from the peritoneal fluid of mice in the infected subgroups were fixed in cold 2.5% buffered glutaraldehyde phosphate, dehydrated using an ascending series of ethanol and examined under scanning electron microscopy (SEM) (JEOL JSM, IT200, Japan) [38].

### IX. Immunological and biochemical studies

Blood samples were collected by cervical incision of each mouse on the 8^th^ day PI. They were centrifuged and serum samples were separated and kept at -20°C to be used for the immunological and biochemical studies.

#### IX.1. Immunological study

The level of IFN-γ was measured in the sera of mice of all subgroups using commercial mouse interferon gamma (IFN-γ) ELISA kit (Chongqing Biospes, China). The assay was carried out according to the manufacturer’s protocol. The detection range of the kit was 31.2–2000 pg/ml.

#### IX.2. Biochemical study

*IX*.*2*.*1*. *Oxidative stress*. Reduced glutathione (GSH) and malondialdehyde (MDA) levels were determined colorimetrically in the sera of mice in all subgroups using commercial kit (Biodiagnostics, Egypt) according to the manufacturer’s instructions.

*IX*.*2*.*2*. *Toxicity*. Sera from mice in the non infected treated subgroups (Ib, Ic and Id) were used to reveal any toxic effect of the treating drugs on liver and kidney functions in comparison to the non-infected non treated subgroup (Ia). Double enzymatic reaction method was used to measure liver enzymes; aspartate transaminase (AST) and alanine transaminase (ALT). While kidney function tests (serum urea and creatinine) were measured by Jaffe reaction method [39].

### X. Statistical analysis of the data

Data were fed to the computer and analyzed using IBM SPSS software package version 20.0. (IBM Corp, Armonk, NY, USA). For continuous data, Shapiro-Wilk test was used to verify the normality of distribution of variables. Quantitative data were described using range (minimum and maximum), mean, standard deviation and median. One way ANOVA test (F-test) was used for normally distributed quantitative variables, to compare between more than two groups, followed by Tukey’s *post Hoc* test for pairwise comparison. Significance of the obtained results was judged at the 5% level (*p* value ≤ 0.05).

## Results

### I. Characterization of MNPs and nZVI

#### I.1. Visual and optical properties

**Fig 1:**
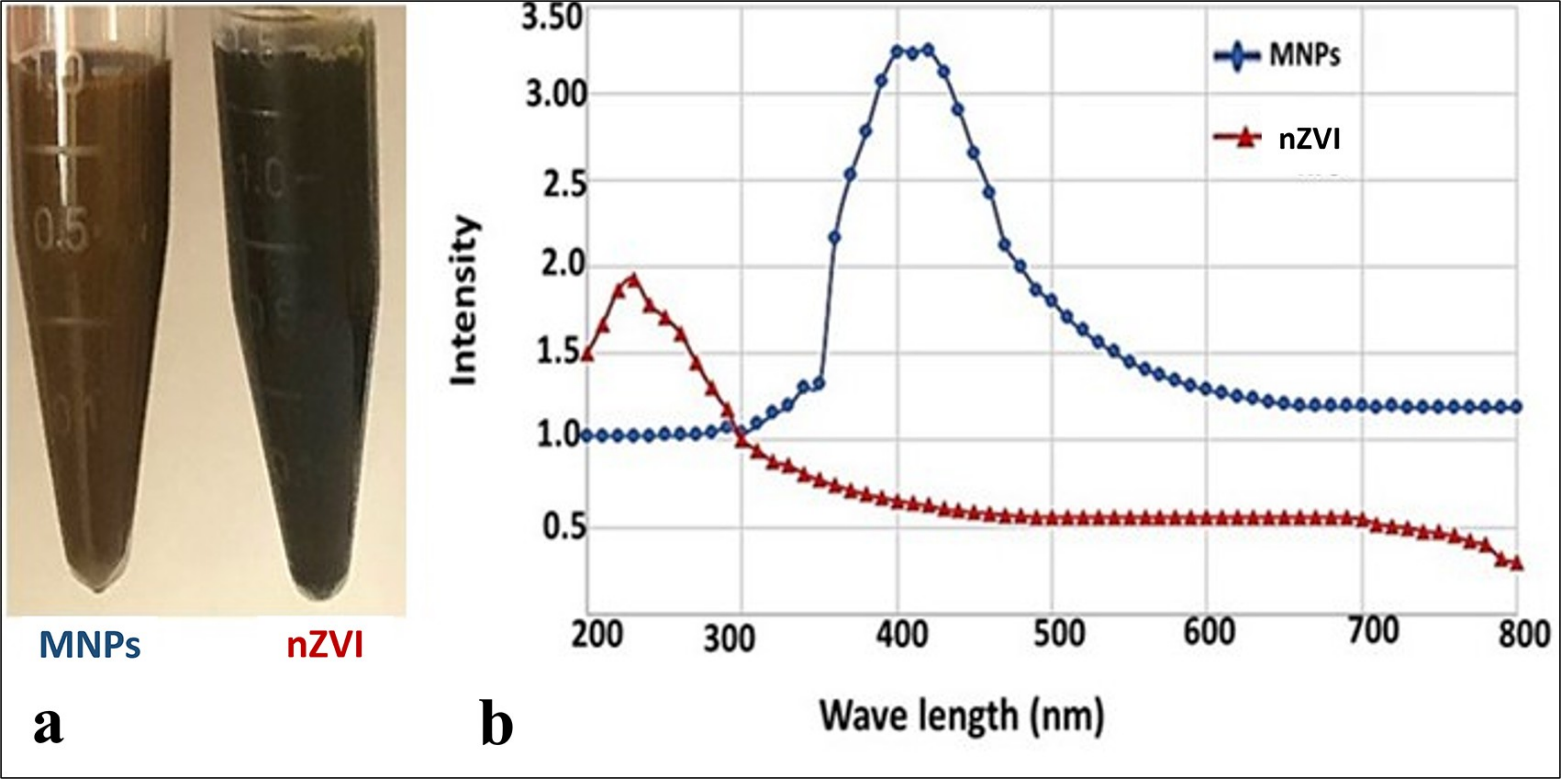
Visual (a) and optical properties (b) of INPs.

The present study sought the eco-friendly fabrication of INPs by *Ochrobactrum sp*. strain CNE2 under nitrate utilization aerobically and anaerobically. Colour changes in the bacterial biomass and its ambient milieu from pale yellow to deep brown/black were affirmed and confirmed by UV-Vis spectrophotometer comparing to the control trials (Fig 1A). The SPR, showed the appearance of single, symmetric, sharp absorption peaks with no tailing at 419 and 233 nm for MNPs and nZVI, respectively (Fig 1B). In agreement with our results, Puthukkara *et al*. [40], stated that the range of 210 to 240 nm in UV-Vis spectra consider being a reliable indicator for the presence of metallic INPs. On the other hand, Mahdavi *et al*. [41], showed that magnetite NPs prepared by *Sargassum muticum* aqueous extract exhibited SPR at 415 nm.

#### I.2. Structural properties

**Fig 2:**
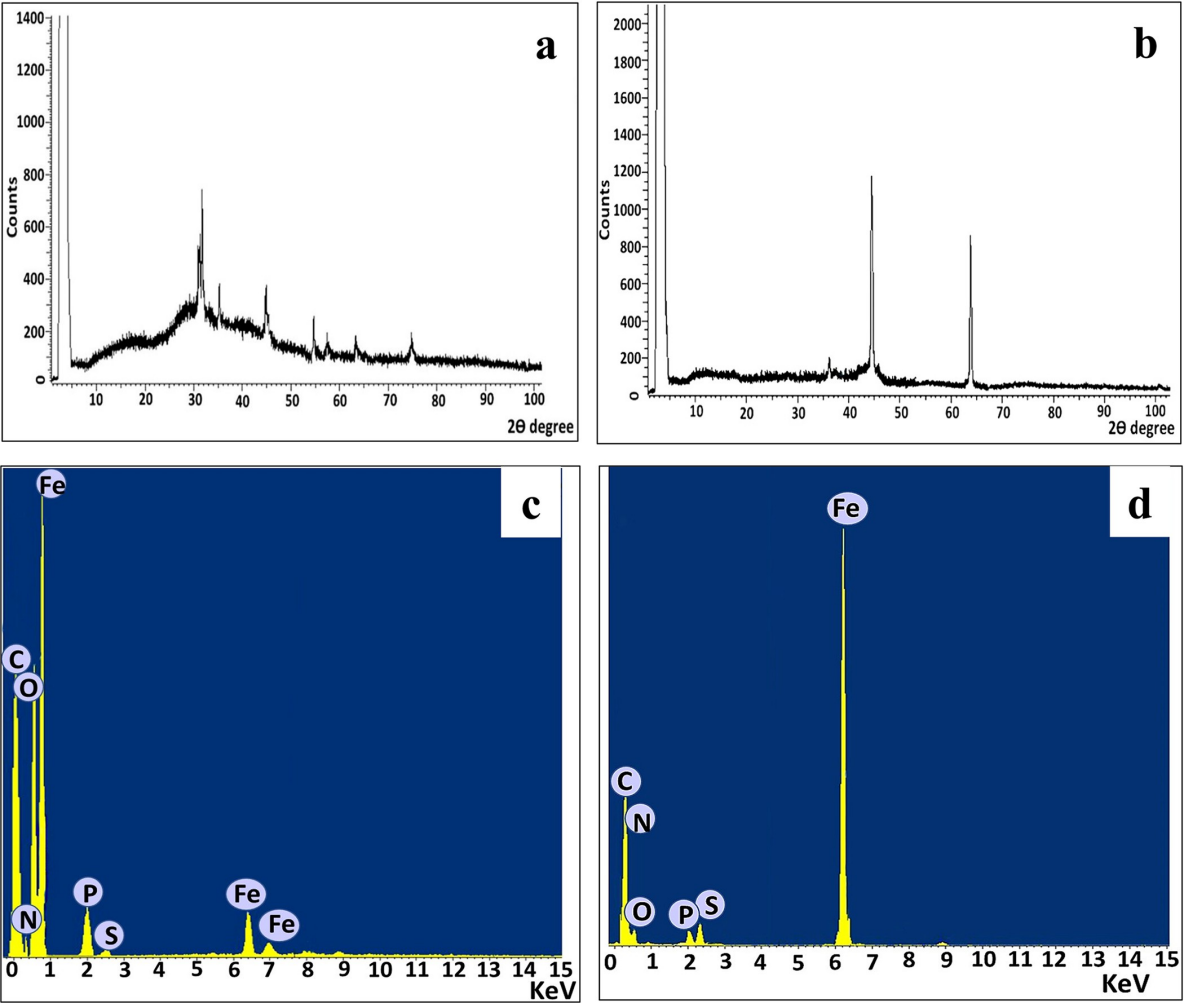
Structural properties of INPs: (a) XRD profile of MNPs; (b) XRD profile of nZVI; (c) EDX pattern of MNPs; (d) EDX pattern of nZVI.

The diffractogram of aerobically synthesized MNPs displayed a series of characteristic peaks at 2θ = 30.9° (220), 35.6° (311), 43.8° (400), 54.5° (422), 57° (511), 63.5° (440) and 74.5° (533); unraveling the formation of orthorhombic phase MNPs (JCPDS no:19–0629) (Fig 2A). However, the anaerobically synthesized nZVI showed two peaks at 2θ = 44.73° and 64.81°, which correspond to Bragg’s reflection values of (110) and (200), respectively. The reflection peaks position and their relative intensities pointed out that the anaerobically synthesized INPs were nZVI and match those of the standard spectrum (JCPDS no: 01-085-1410). Notably, small peak was observed at 2θ = 36.11°, which corresponds to (111) plane of FeO (JCPDS no: 772355) (Fig 2B). The presence of this peak in low intensity revealed the oxidation of nZVI surface during extraction, washing and processing step, which agreed with the results obtained by chemical and phyco-synthesis methods [42]. Undoubtedly, the presence of sharp and obviously distinguished peaks in XRD diffractograms of both aerobically and anaerobically synthesized INPs indicated the crystalline nature of them with the lowest surface energy [43].

The elemental profile of MNPs prepared aerobically by strain CNE2 confirmed that iron and oxygen were the major ingredients in the examined material. The iron main peaks were noticed at binding energies of 0.8, 6.3 and 7.0 keV with atomic percentages of 57.11%; a remarkable signal at 0.5 KeV with atomic percentage of 29.75% was identified for oxygen (Fig 2C). While predominant peak was observed at range of 6–7 keV with atomic percentages of 77.3% that is related to iron binding energy in nZVI sample (Fig 2D). Additional small peak was observed in anaerobically synthesized nZVI at binding energy of oxygen (0.5 keV) with atomic percentages of 1.5% (Fig 2D). In addition, the peaks closer to 0.27, 0.3, 2.0 and 2.3 keV are associated to the binding energies of carbon, nitrogen, phosphorous and sulfur, correspondingly with considerable atomic percentages (Fig 2C and 2D). This result agreed with that obtained by Rahman *et al*., [44] who reported that association of these elements with NPs was routinely observed in green synthesis methods.

#### I.3. Morphological properties

**Fig 3:**
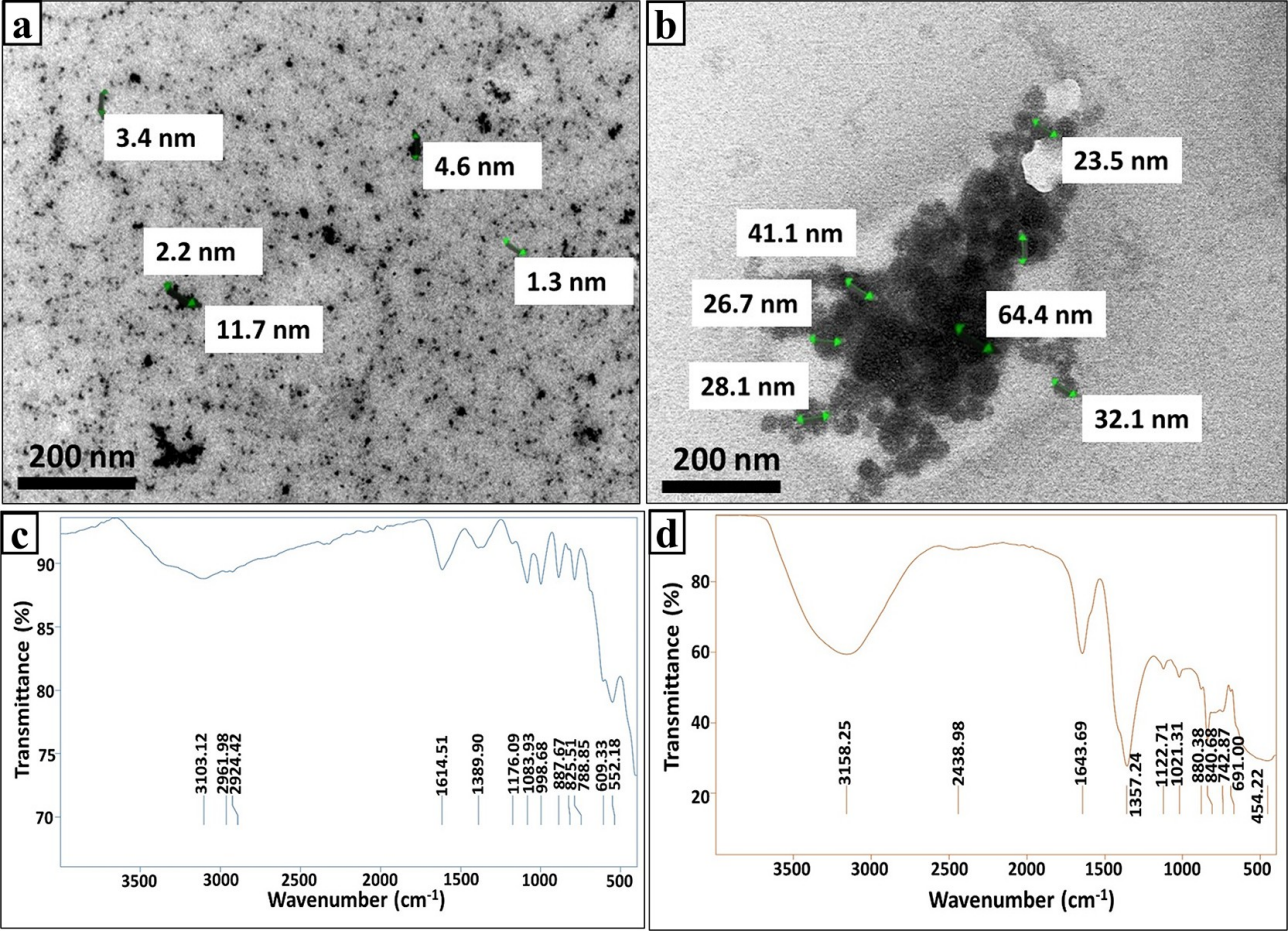
Morphological and functional properties of INPs: (a) TEM of MNPs; (b) TEM of nZVI; (c) FTIR spectrum of MNPs; (d) FTIR spectrum of nZVI.

The microstructure analysis of biogenic INPs, which include morphology, size and their dispersion uniformity, was studied by TEM. The TEM micrographs displayed textural heterogeneity and disparity between MNPs and nZVI; implying the obvious influence of incubation conditions. MNPs that were synthesized aerobically showed numerous, tiny, roughly globular, homogenously distribute with no apparent aggregation and ranged in size from 1.3–11.7 nm (Fig 3A). Whereases, nZVI appeared as large uniform spheres oscillated in their size between 23.5 and 64.4 nm aggregated in nanoclusters (Fig 3B). Such morphological differences between MNPs and nZVI was found previously by Nadeem *et al*. [45], who assigned that several parameters were involved microbial chemistry, metal type, reaction conditions, interaction mechanism and entire physical state of the reaction.

#### I.4. Functional properties

As depicted by Fig 3C and 3D the presence of some intensive bands in FTIR patterns of both INPs. Initially, the vibration bands at 3103 and 3158 cm^-1^ were attributed to stretching vibrations of O–H groups of water molecule that was physically adsorbed on INPs [46]. Meanwhile, the wave numbers of 2961 and 2924 cm^-1^ corresponded to the stretching vibration of C-H [47]. Whereas, the bands at 2438 cm^-1^ implied the existence of C = O bonds related to proteins [48]. Besides, the peaks at 1614 and 1643 cm^-1^ are ascribed to amide-I/amide-II linkages, reflecting the conjugation of proteins [47]. The spectral bands at 1357 and 1389 cm^-1^ are assigned to symmetric stretch carboxyl groups (–COOH) [49]. Whereas the bands at 1122 and 1176 cm^-1^ implied the existence of amines C–N [47]. However, the peaks of 1083 and 1021 cm^-1^ could be attributed to the symmetric C–O vibration [41]. The band at 998 cm^-1^ indicated the occurrence of PO_4_^3-^ group [50]. Regarding the peaks at 887, 880, 840, 825, 788, 742 cm^-1^, they could be assigned probably to C-H bending vibrations [51]. Interestingly, the formation of MNPs was confirmed through some distinctive spectral bands such as 552 and 609 cm^-1^, which are assigned to Fe–O bond in MNPs [41]. Broadly, it is vividly evident the association of Fe with several functional groups, which supported the former results of EDX.

#### I.5. Particles surface properties

**Fig 4:**
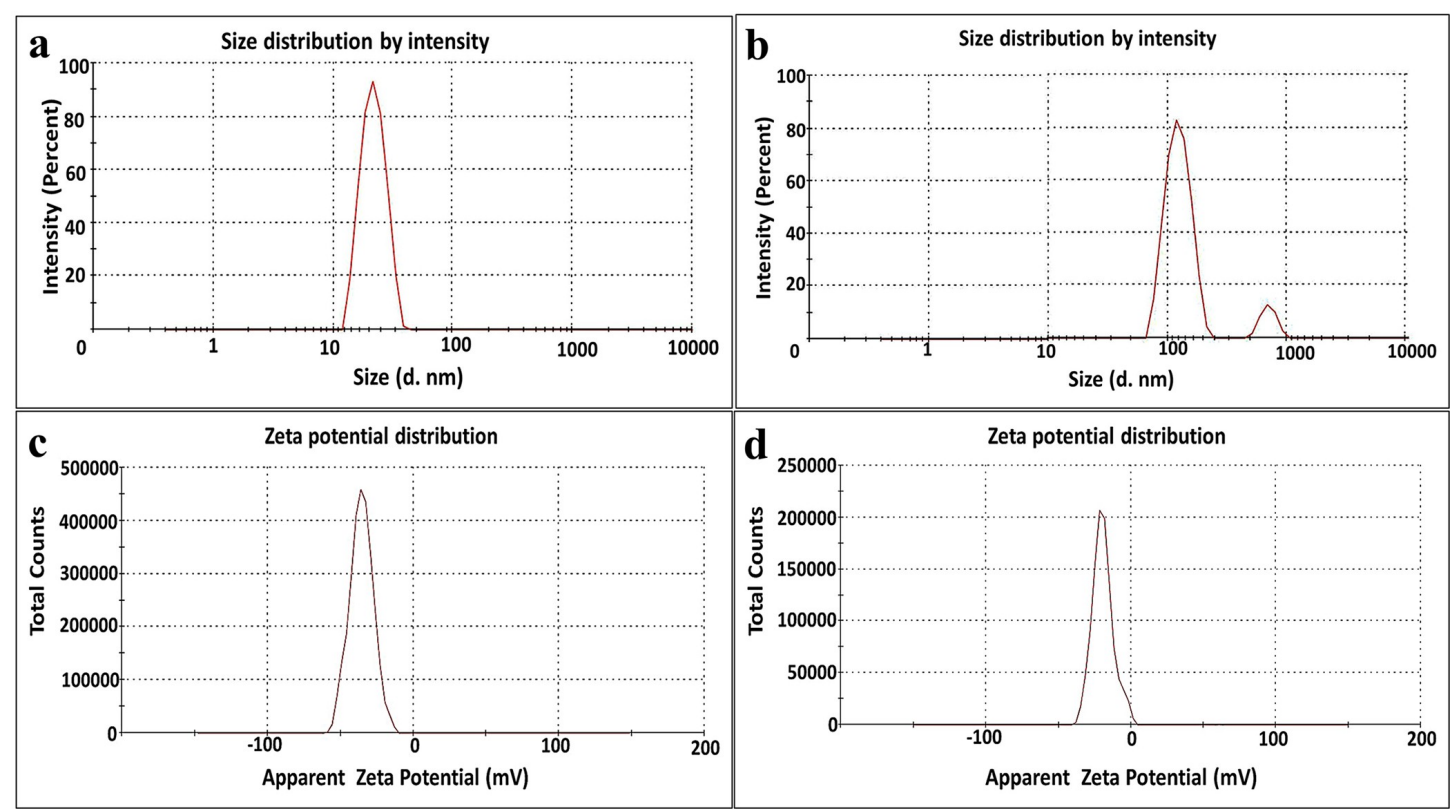
Particle surface properties of INPs: (a) Particle size distribution of MNPs; (b) Particle size distribution of nZVI; (c) Zeta potential of MNPs; (d) Zeta potential of nZVI.

The particle size distribution curve of MNPs denoted that the particle size assessed by 46.82 nm (99.72%) with a standard deviation of 19.02% (Fig 4A), while the hydrodynamic size of nZVI recorded 126.24 nm (89.5%) and 912.1 nm (11.5%) with a standard deviation of 20.62% and 9.83%, respectively (Fig 4B). Remarkably, PDI values implied higher monodispersity of smaller MNPs (0.219 nm) in a homogenous distribution than that exhibited by larger nZVI (0.433 nm). Further, ZP of MNPs and nZVI was -35.5 and -20.9 mV, respectively, revealing good and moderate stability of MNPs and nZVI, correspondingly, according to colloid stability ranking based on ZP as stated by Vishwakarma [52]. Notably, the negative sign of ZP pointed out to the existence of negatively charged matrix of bacterial biomolecules [53].

### II. Parasitological study

#### II.1. Mortality rate

There was statistically non-significant difference in mortality rate among all studied subgroups (*P > 0*.*05*). Death of one mouse was recorded in infected non treated subgroup IIa on the 7^th^ day PI with a mortality rate of 14.28%, whereas no mortalities among mice in other studied subgroups till the sacrifice time (8^th^ day PI) were observed.

#### II.2. Parasite count

As shown in Table 1, a statistically significant reduction in the mean number of tachyzoites in the peritoneal fluid and liver impression smears was detected in all infected treated subgroups (IIb, IIc and IId) compared to the infected non treated subgroup IIa (*P < 0*.*001*). Oral administration of MNPs and nZVI to the infected mice resulted in a statistically significant reduction in mean tachyzoite count in peritoneal fluid and liver impression smears (12.83 ± 0.61 and 0.88 ± 0.08) and (99 ± 9.23 and 3.35 ± 0.36) for the two subgroups, respectively, compared to a mean of (403.1 ± 82.54 and 10.82 ± 2.16) for the infected non treated control subgroup (*P < 0*.*001*). On comparing the effect of MNPs and nZVI with that of SPI on tachyzoite count in peritoneal fluid and liver impression smears, a statistically significant difference was only recorded between nZVI and SPI (P < 0.001). While the comparison between the biogenic MNPs and nZVI revealed a statistically significant difference in mean parasite burden in peritoneal fluid and liver impression smears in favour of MNPs (*P < 0*.*001*).

**Table 1:** The effect of SPI, MNPs and nZVI on the tachyzoites’ burden in peritoneal fluid and liver impression smears of the infected treated subgroups of mice compared to the infected non-treated control.

Subgroup Tachyzoite count	Non treated (IIa)	SPI-treated (IIb)	MNPs-treated (IIc)	nZVI-treated (IId)	F	p
**Peritoneal fluid x10** ^**4**^						
Median (Min.–Max.)	394 (312–543.5)	29.5 (27.5–30.5)	12.8 (12–13.5)	96.3 (88.5–114)	115.116*	<0.001*
Mean ± SD.	403.1 ± 82.54	29.2 ± 1	12.83 ± 0.61	99 ± 9.23		
%R		**92.76**	**96.82**	**75.44**		
p_0_		<0.001*	<0.001*	<0.001*		
Significance		p_1_ = 0.903, p_2_ = 0.040*, p_3_ = 0.009*				
**Liver impression smears**						
Median (Min.–Max.)	11.3 (7.4–13.4)	1.5 (1.2–1.6)	0.90 (0.80–1.0)	3.4 (2.8–3.7)	105.043*	<0.001*
Mean ± SD.	10.82 ± 2.16	1.45 ± 0.14	0.88 ± 0.08	3.35 ± 0.36		
%R		**86.60**	**91.87**	**69.04**		
p_0_		<0.001*	<0.001*	<0.001*		
Significance		p_1_ = 0.807, p_2_ = 0.033*, p_3_ = 0.005*				

% R: Percentage of reduction in each of the studied subgroups relative to infected non treated control

F: F for One way ANOVA test, used in comparison between more than two groups

Post Hoc test (Tukey) is used in pairwise comparisons

p: p value for comparing between the subgroups

p_0_: p value for comparing between infected non treated subgroup (IIa) and each other infected treated subgroups

p_1_: p value for comparing between SPI-treated subgroup (IIb) and MNPs-treated subgroup (IIc)

p_2_: p value for comparing between SPI-treated subgroup (IIb) and nZVI**-**treated subgroup (IId)

p_3_: p value for comparing between MNPs-treated subgroup (IIc) and nZVI**-**treated subgroup (IId)

*: Statistically significant at p ≤ 0.05

### III. Ultrastructural study

**Fig 5:**
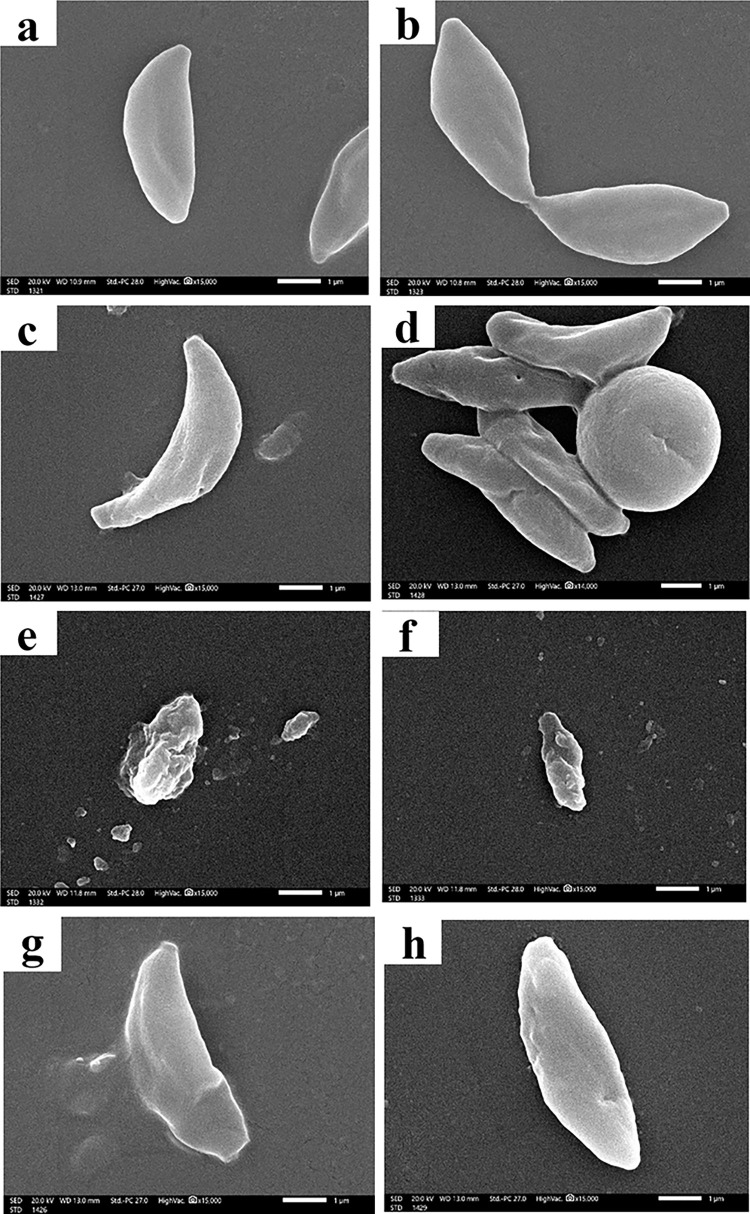
SEM images of *T*. *gondii* tachyzoites recovered from peritoneal fluid of infected mice: (a & b) Non treated tachyzoites showing typical crescent shape and smooth body with intact regular membrane (x15,000); (c & d) SPI-treated tachyzoites showing minimal changes in the form of mild irregularities and few dimples on the crescent shape surface (x15,000); (e-f) MNPs-treated tachyzoites showed (e) markedly distorted body with multiple deep ridges and furrows (x15,000); (f) shrunken body and obvious protrusions (x15,000); (g-h) nZVI-treated tachyzoites showed (g) distortion of the posterior end with plasma membrane erosions and ulcerations (x15,000); (h) external protrusions and dimples (x15,000).

Results of SEM examination are shown in Fig 5. Tachyzoites of mice in the infected non treated subgroup (IIa) appeared generally crescent in shape with completely smooth regular surfaces (Fig 5A and 5B). Whereas treated tachyzoites showed variable surface changes. Most tachyzoites of SPI-treated subgroup (IIb) preserved their crescent shape and the surfaces of most of them were nearly smooth with mild irregularities and few dimples (Fig 5C and 5D). On the other hand, severe and profound morphological changes were detected in tachyzoites of MNPs-treated subgroup (IIc). Most of them appeared shrunken and obviously distorted. Furthermore, the tachyzoite surfaces exhibited marked irregularities with numerous ridges and protrusions. Additionally, noticeable distortion in the apical region was observed in some tachyzoites (Fig 5E and 5F). Comparable but milder surface changes were observed in nZVI-treated subgroup (IId). Despite keeping their crescent shape, some tachyzoites in nZVI-treated subgroup showed surface protrusions and multiple dimples, others showed irregular surface ridges, erosions and distorted posterior end (Fig 5G and 5H).

### IV. Immunological and biochemical studies

#### IV.1. Immunological study

**Fig 6:**
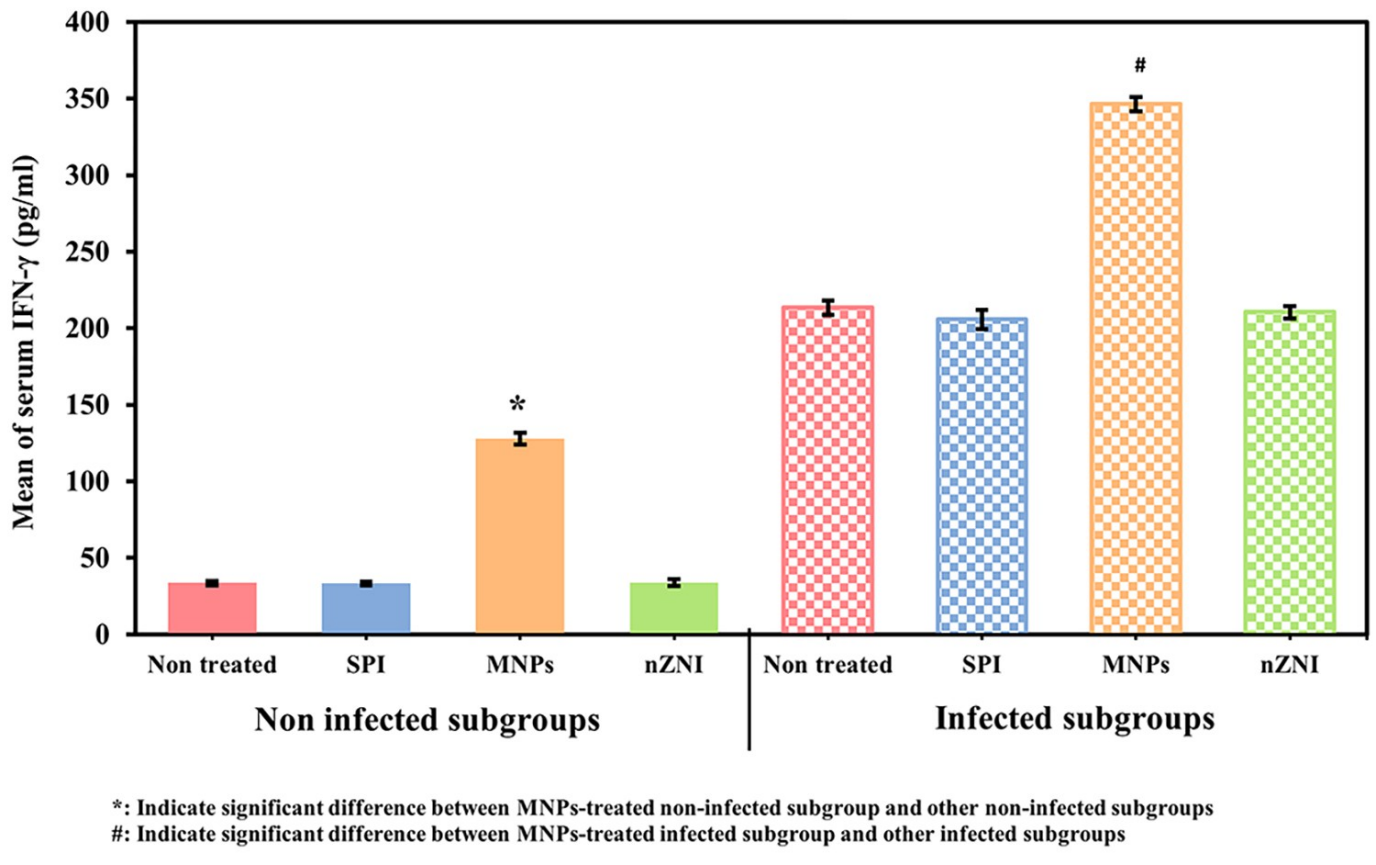
Serum IFN-γ levels among the different studied subgroups.

As regards the non infected subgroups, a statistically significant increase in the mean values of serum IFN-γ was recorded in MNPs-treated subgroup (Ic) compared to other subgroups either treated or not. Generally, during the course of infection, the mean levels of INF-γ highly increased in all infected subgroups whether treated or not. The most statistically significant increase in the serum levels of INF-γ among all infected subgroups was only recorded in MNPs-treated infected subgroup (IIc). Whereas the infected subgroups treated with SPI and nZVI showed non-statistically significant difference in the mean levels of INF-γ in comparison with the infected non treated subgroup (Fig 6). Detailed serological results were shown in S1 Table.

#### IV.2. Biochemical study

*IV*.*2*.*1*. *Oxidative stress*. There was no statistically significant change in sera levels of MDA in non infected treated subgroups (Ib, Ic and Id) in comparison to non infected non treated subgroup (Ia). On the contrary, all infected treated subgroups (IIb, IIc and IId) showed a statistically significant decrease in their serum levels of MDA when compared to the infected non treated subgroup (IIa). The least statistically significant reduction in MDA was noticed in infected nZVI-treated subgroup (IId), followed by infected SPI-treated subgroup (IIb) with slight non-significant difference. While MNPs-treated subgroup achieved a profound statistically significant decrease in MDA in comparison to either SPI-treated or nZVI-treated subgroups (Table 2).

**Table 2:** The effect of SPI, MNPs and nZVI on the serum MDA level in nmol/ml among all studied subgroups compared to their controls.

		Serum MDA				
**Non infected subgroup**	**Non treated (Ia)**	**SPI-treated (Ib)**	**MNPs-treated (Ic)**	**nZVI-treated (Id)**	**F**	**p**
Median (Min.–Max.)	6.41 (5.89–6.95)	6.90 (6.25–7.26)	6.50 (6.20–7.31)	6.77 (6.70–7.24)	2.713	0.072
Mean ± SD	6.36 ± 0.41	6.84 ± 0.34	6.61 ± 0.43	6.89 ± 0.23		
**Infected subgroup**	**Non treated (IIa)**	**SPI-treated (IIb)**	**MNPs-treated (IIc)**	**nZVI-treated (IId)**	**F**	**p**
Median (Min.–Max.)	22.82 (18.99–23.60)	12.69 (11.50–13.15)	7.95 (7.80–8.20)	13.28 (12.13–13.70)	210.893*	<0.001*
Mean ± SD	22.08 ± 1.83	12.56 ± 0.55	7.98± 0.15	13.12 ± 0.54		
p_0_		<0.001*	<0.001*	<0.001*		
Significance		p_1_<0.001*, p_2_ = 0.770, p_3_<0.001*				

F: F for One way ANOVA test, used in comparison between more than two groups

Post Hoc test (Tukey) is used in pairwise comparisons

p: p value for comparing between the subgroups

p_0_: p value for comparing between infected non treated subgroup (IIa) and each other infected treated subgroups

p_1_: p value for comparing between SPI-treated subgroup (IIb) and MNPs-treated subgroup (IIc)

p_2_: p value for comparing between SPI-treated subgroup (IIb) and nZVI**-**treated subgroup (IId)

p_3_: p value for comparing between MNPs-treated subgroup (IIc) and nZVI**-**treated subgroup (IId)

*: Statistically significant at p ≤ 0.05

All non-infected treated subgroups (Ib, Ic and Id) revealed no statistically significant difference in the mean sera levels of GSH when compared to non infected non treated subgroup (Ia). On the other hand, there was a statistically significant increase in the mean serum levels of GSH noticed in all infected treated subgroups (IIb, IIc and IId) compared to the infected non treated subgroup (IIa). Meanwhile, there was a pronounced significant increase in GSH observed in MNPs-treated subgroup (IIc) as compared with the other infected treated subgroups (IIb and IId) (Table 3).

**Table 3:** The effect of SPI, MNPs and nZVI on the serum GSH level in mg/dl of among all studied subgroups compared to their controls.

		Serum GSH				
**Non infected subgroup**	**Non treated (Ia)**	**SPI-treated (Ib)**	**MNPs-treated (Ic)**	**nZVI-treated (Id)**	**F**	**p**
Median (Min.–Max.)	2.63 (2.22–2.98)	2.86 (2.65–3.12)	2.67 (2.45–3.10)	2.84 (2.39–3.12)	0.957	0.432
Mean ± SD	2.62 ± 0.26	2.88 ± 0.20	2.73 ± 0.25	2.77 ± 0.33		
**Infected subgroup**	**Non treated (IIa)**	**SPI-treated (IIb)**	**MNPs-treated (IIc)**	**nZVI-treated (IId)**	**F**	**p**
Median (Min.–Max.)	1.22 (0.98–1.50)	2.51 (2.00–2.85)	8.89 (8.35–9.21)	4.52 (3.95–4.73)	740.935*	<0.001*
Mean ± SD	1.23 ± 0.24	2.44 ± 0.30	8.83 ± 0.30	4.40 ± 0.34		
p_0_		<0.001*	<0.001*	<0.001*		
Significance		p_1_<0.001*, p_2_<0.001*, p_3_<0.001*				

F: F for One way ANOVA test, used in comparison between more than two groups

Post Hoc test (Tukey) is used in pairwise comparisons

p: p value for comparing between the subgroups

p_0_: p value for comparing between infected non treated subgroup (IIa) and each other infected treated subgroups

p_1_: p value for comparing between SPI-treated subgroup (IIb) and MNPs-treated subgroup (IIc)

p_2_: p value for comparing between SPI-treated subgroup (IIb) and nZVI**-**treated subgroup (IId)

p_3_: p value for comparing between MNPs-treated subgroup (IIc) and nZVI**-**treated subgroup (IId)

*: Statistically significant at p ≤ 0.05

*IV*.*2*.*2*. *Toxicity*. Levels of liver enzymes (AST and ALT) and kidney function tests (urea and creatinine) in the sera of non infected mice treated with SPI, MNPs and nZVI did not reveal any statistically significant difference in comparison with the non infected non treated mice (Table 4).

**Table 4:** The effect of SPI, MNPs and nZVI on the level of ALT, AST, urea and creatinine in sera of mice of non infected treated subgroups compared to their non treated control.

Subgroup Biomarker	Non treated (Ia)	SPI-treated (Ib)	MNPs-treated (Ic)	nZVI-treated (Id)	F	p
**ALT (IU/L)**						
Median (Min.–Max.)	26.5 (25–28)	26.2 (24.3–29)	26.8 (24.7–28.5)	27.3 (26.1–30)	0.705	0.560
Mean ± SD	26.54 ± 1.29	26.44 ± 1.58	26.71 ± 1.53	27.53 ± 1.39		
**AST (IU/L)**						
Median (Min.–Max.)	22.9 (21–25.8)	23.8 (22.3–26.5)	24.3 (20.7–25.6)	24.1 (22.16–27.0)	0.459	0.714
Mean ± SD	23.13 ± 1.84	24.10 ± 1.84	23.76 ± 1.81	24.28 ± 1.81		
**Urea (mg/dl)**				
Median (Min.–Max.)	14 (10.7–16)	15.5 (11.5–16.5)	13.2 (11–15.7)	14.2 (12.9–17.1)	0.847	0.484
Mean ± SD	13.9 ± 2	14.8 ± 2	13.3 ± 1.6	14.7 ± 1.9		
**Creatinine (mg/dl)**						
Median (Min.–Max.)	0.70 (0.54–0.88)	0.73 (0.65–0.90)	0.79 (0.57–0.92)	0.70 (0.65–0.95)	0.387	0.763
Mean ± SD	0.70 ± 0.13	0.75 ± 0.09	0.77 ± 0.13	0.75 ± 0.12		

F: F for One way ANOVA test, used in comparison between more than two groups

p: p value for comparing between the subgroups

## Discussion

Toxoplasmosis is a global health problem caused by the zoonotic parasite, *T*. *gondii* [2]. The gold standard treatment of toxoplasmosis has recently come under scrutiny because of their serious adverse effects and emergence of resistant strains [8]. The discovery of safe and effective natural compounds for treatment of *T*. *gondii* infection seems to be a priority and this is where nanobiotechnology is having a greater chance [54]. Selection of biogenic INPs in the current study was based merely on their unique physicochemical and biological properties (e.g., antimicrobial, antischistosomal activity, etc.), which exceed those fabricated by physicochemical methods [22,25,30]. Herein, the biosynthesis of two types of INPs was successfully fulfilled by the dint of metabolic capability of bionanofactory *Ochrobactrum sp*. *CNE2*. Via cascade of steps, the biofabrication mechanism fall under the umbrella of oxidation–reduction (redox) reaction. In fact, the nitrate reductase and conjugated electron shuttling molecules may shuttle electrons to the metal ions, leading ultimately to the formation of iron in its nanoform [55]. However, the discripancy in final end product identity could be assigned to the physiological behavior of bacterial cells during growth cycle which express different enzymatic systems in both conditions. Namely, under conditions of oxygen availability, oxide form of INPs were synthesized by the catalysis of periplasmic nitrate reductase [56,57]. On the other hand, in anoxic conditions (i.e., complete absence of oxygen), nZVI was synthesized by the action of respiratory nitrate reductase that enhances the anerobic respiration of nitrate and subsequent generation of the proton motive force [34,41].

Via this bottom-up approach, the biogenic INPs exerted several advantageous features such as; crystalline nature, stability, charge and surface coating [43]. Remarkably, coating the surface of INPs with bacterially-originated functional groups; C = O, C-N, C-H,–OH,–COOH, amide, and PO_4_^3-^, in the same stage of bioreduction, played a vital role in maintaining self-functionalization, stability and facilitating their entry into living cells and bloodstream [34,44]. The negatively charged bacterial matrix biomolecules; sugar-phosphate backbone of nucleic acid residues and amino acids as aspartate and glutamate, act as capping agents that maintain particles functionality and accelerate their interactions with cells [53].

Upon employing both INPs as antitoxoplasmic agents, a potent effectiveness on the tachyzoites was observed as revealed by parasitological and ultrastructural results. However, MNPs possess higher antitoxoplasmic activity than nZVI and SPI evidenced by the highest reduction in count, remarkable shrinkage in size and extensive damage to the surface of tachyzoites. This can be related to the characteristic physicochemical properties of MNPs such as size, surface area, PDI and ZP. Our findings agreed with Zaki *et al*., [34], who observed that MNPs were more efficient than nZVI in microbial growth suppression in all examined water samples. Similarly, Younis *et al*., [25], reported that MNPs showed privilege over nZVI in reducing adult *S*. *mansoni* worm burden and liver granulomata number. The smaller MNPs (less than 30 nm) have exponentially more number of atoms on their surface than the larger nZVI which help them bind and cross the membranes, hence take part in subcellular reactions [54]. As documented, the reduction in size of NPs is accompanied by more reactive surface area and subsequently induction of higher amount of reactive oxygen species (ROS) [58]. In addition, the smaller MNPs with high monodispersity (PDI of 0.219) tend to homogenously distribute than larger nonuniform nZVI (with PDI of 0.433). According to colloid stability ranking stated by Vishwakarma *et al*., [52], MNPs with ZP -35.5 mV (higher than ± 30 mV) exhibited better long-term stability than nZVI (-20.9 mV) due to increased electro-static repulsion.

Despite the obvious reduction in parasite burden, SPI showed minimal effect on surface of tachyzoites, supporting earlier observation by Gamea *et al*., [59] who reported minimal surface irregularities of the tachyzoites. This could be attributed to the mechanism of action of SPI, as it binds to the 50S ribosomal subunit, preventing peptide chains elongation, and thus, inhibition of protein synthesis [60].

IFN-γ is the keystone cytokine of the protective cellular immune response against *T*. *gondii* infection that prevents massive parasitaemia [61]. In the current study, sera IFN-γ levels were obviously increased in noninfected MNPs-treated mice (subgroup Ic) as compared to other noninfected subgroups. Our results were in accordance with Chen *et al*., who reported increased serum IFN-γ level in normal ICR mice IV injected with chemically synthesized MNPs [62]. Noticeably, IFN-γ sera levels were increased following the *Toxoplasma* infection (subgroup IIa). Our results were in accordance with Gaafar *et al*., [10] and Hegazi *et al*., [63] who reported that a strong cellular immune response was elicited by high IFN-γ production during acute and chronic toxoplasmosis, respectively. Although, the IFN-γ production is apparently blocked in the infected cells, rapid growth of the *T*. *gondii* is contained by a vigorous IFN-γ-dependent immune response by surrounding non infected immune cells [61,64]. However, following oral administration of INPs, the highest sera levels of IFN-γ were achieved by infected mice treated with MNPs (subgroup IIc) as a result of their enhancement of immunity. These results demonstrates that the significant reduction in parasite load and marked ultrastructural changes in tachyzoites in the infected mice treated with MNPs (subgroup IIc) might be linked to the augmentation of the protective IFN-γ-dependent cell-mediated immune response which led to the control of infection.

Determination of MDA and GSH sera levels in *T*. *gondii* infection is a reliable indicator for follow up and control of treatment [65]. In infected mice of subgroup (IIa), serum MDA was obviously elevated, whereas, antioxidant GSH serum level was markedly decreased. Similarly, Nazarlu *et al*., [66] reported marked increment in MDA and decline in GSH concentration in the serum of rats infected with *T*. *gondii*. The decrease in GSH, verified that the antioxidant defence system is overwhelmed by the amount of MDA produced in response to the *T*. *gondii* infection [67].

On the other hand, both INPs markedly decreased MDA and increased GSH sera levels after *T*. *gondi* challenge (subgroups IIc and IId). Biocompatible INPs is efficiently internalised by tachyzoites, metabolized as the endogenous iron and degraded by hydrolysis into free iron ions which converted the mitochondrial hydrogen peroxide to form a highly reactive hydroxyl radicals via Fenton reaction [68,69]. Such ROS were responsible for the induction of oxidative stress that damaged the protein, DNA, cell membrane and other vital enzymes eventually leading to apoptosis [58]. In addition, oxidative stress could also favor pores formation in the mitochondria, thus impairing the organelle function [70]. Thus, biogenic nanoscaled iron, MNPs and nZVI, had the potential to be used as antioxidants, by restoration of the GSH level that quenched ROS product, MDA, associated with the *T*. *gondii* infection [71]. MNPs had major potential to undergo oxidation and cause higher levels of antioxidants than nZVI. This could be attributed to their morphology, size, biocompatibility, superparamagnetic, catalytic and biochemical properties [69].

There are concerns on potential nanotoxicity associated with some metal NPs, as silver NPs, even at low concentration and their harmful accumulation in vital organs [72]. The safety of biogenic INPs, MNPs and nZVI, was proved by measuring the liver and renal function biomarkers in serum of mice [73]. These data were consistent with El-Bahr *et al*., [74], who indicated that oral administration of MNPs biosynthesized from *Petroselinum crispum* leaf extract did not induce any hepatic nor renal toxicity in male albino rats. Similarly, Tavakoli *et al*., [75] demonstrated that intraperitoneal injection of nZVI synthesized by *Myrtus communis* did not increase serum level of hepatic enzymes.

## Conclusions

Biofabricated INPs, MNPs and nZVI, can serve as highly potent nanotherapeutic agents affording high levels of safety with antitoxoplasmic activity. The promising antitoxoplasmic activity of biogenic INPs appeared to be related to two main interplaying protective mechanisms; immunological, represented by IFN-γ production, and antioxidant mechanisms. Biosynthesized INPs, especially MNPs, may herald a revolution in pharmacological therapy for *T*. *gondii* infections. Further verification of the underlying pharmacodynamics and kinetics of the biosafe MNPs is still needed to be studied.

## Supporting information

S1 Table Supporting data for Fig 6. (DOCX)
