# Supplementary material for: Therapeutic and antioxidant potential of bionanofactory Ochrobactrum sp.-mediated magnetite and zerovalent iron nanoparticles against acute experimental toxoplasmosis
Source: PLoS Negl Trop Dis. 2023 Oct 6;17(10):e0011655. doi: 10.1371/journal.pntd.0011655 (PMC10558077; doi:10.1371/journal.pntd.0011655)
Supplement: S1 Table — (DOCX) [file pntd.0011655.s001.docx]

**S1 Table: The effect of SPI, MNPs and nZVI on the serum IFN-γ level in pg/ml among all studied subgroups compared to their controls**

| **Non infected subgroup** | **Non treated  (Ia)** | **SPI-treated  (Ib)** | **MNPs-treated (Ic)** | **nZVI-treated (Id)** | **F** | **p** |
| --- | --- | --- | --- | --- | --- | --- |
| Median (Min. – Max.) | 33.8 (31.5 – 35) | 33.1 (31.5 – 34.7) | 128.5 (121.5 – 132.4) | 34.1 (30 – 36.4) | 2220.47^*^ | <0.001^*^ |
| Mean ± SD | 33.5 ± 1.4 | 33.1 ± 1.3 | 127.8 ± 4 | 33.8 ± 2.2 |  |  |
| p_0_ |  | 0.990 | <0.001^*^ | 0.999 |  |  |
| Significance |  | p_1_<0.001^*^, p_2_=0.968, p_3_<0.001^*^ | | |  |  |
| **Infected subgroup** | **Non treated  (IIa)** | **SPI-treated (IIb)** | **MNPs-treated  (IIc)** | **nZVI-treated  (IId)** | **F** | **p** |
| Median (Min. – Max.) | 214.6 (205.8 – 218.4) | 207.1 (198 – 211.8) | 347 (339.3 – 351.5) | 210.5 (205.3 – 216.2) | 1142.345^*^ | <0.001^*^ |
| Mean ± SD | 213.3 ± 4.7 | 205.6 ± 6.1 | 346.2 ± 4.6 | 210.3 ± 4.2 |  |  |
| p_0_ |  | 0.062 | <0.001^*^ | 0.721 |  |  |
| Significance |  | p_1_<0.001^*^, p_2_=0.382, p_3_<0.001^*^ | | |  |  |

F: F for One way ANOVA test, used in comparison between more than two groups

Post Hoc test (Tukey) is used in pairwise comparisons

p: p value for comparing between the subgroups

p_0_: p value for comparing between non-treated subgroup (Ia or IIa) and other treated subgroup

p_1_: p value for comparing between SPI-treated subgroup and MNPs-treated subgroup (Ib and Ic) or (IIb and IIc)

p_2_: p value for comparing between SPI-treated subgroup and nZVI**-**treated subgroup (Ib and Id) or (IIb and IId)

p_3_: p value for comparing between MNPs-treated subgroup and nZVI**-**treated subgroup (Ic and Id) or (IIc and IId)

*: Statistically significant at p ≤ 0.05
